# Notes and News

**Published:** 1895-04-06

**Authors:** 


### Notes and News.

Collected and Communicated.

We are glad to notice that the new secretary of the York County Hospital is determined to keep his institution up to the times. Many applications having been made for the loan of an ambulance from the city and surrounding suburbs, the committee have decided to purchase one. After making thorough inquiries as to the best apparatus, the "Viaduct Ambulance Litter" has been determined upon. This is that adopted by the Hospitals Association Ambulance Branch for the London Street Ambulance Service, and is made by Alfred Carter, of Holborn Viaduct. The ambulance will now be kept ready for all emergencies at the hospital.

The annual festival dinner of the British Orphan Asylum, Slough, is announced to take place on May 10th, at the Hotel Metropole, when Bishop Barry, D.D., will preside. A special appeal for support is being issued this year, as the directors have unfortunately to report a considerable falling off in subscriptions, while few legacies or donations have been received, with the result that there is a large deficiency in the usual income, and it has been necessary to encroach on the reserve fund and also to borrow. The work done by the British Orphan Asylum is so excellent that its needs should only be known for sympathy and help to be at once enlisted. For seventy years it has carried on its beneficent labours for the good of those children who are "deprived of the natural protection of parentage," especially touching a "class which beyond most others has to suffer in silence," by admitting the children of those who have been reduced to poverty from respectability. More than 1,800 children have thus been given a fair start in life, and there are nearly 200 now in the institution. We hope a special effort will be made by many to be present at the annual dinner, and to contribute to the funds of so deserving a charity.

The late Earl of Moray has left a legacy of £50,000 to the Edinburgh Royal Infirmary.

A fresh outbreak of the plague is reported from Kowloon, a portion of the colony of Hong Kong on the mainland, just across the harbour.

The vacancy on the staff of the Middlesex Hospital created by the death of Mr. J. W. Hulke has been filled by the unanimous election of Mr. Andrew Clark, F.R.C.S.

On Wednesday, March 27th, a complimentary dinner was given at the Criterion to Sir John Erichsen, Sir Russell Reynolds, and Sir John Williams by their colleagues and pupils at University College Hospital. The chair was taken by Lord Reay, Vice-President of University College, and some hundred and eighty guests were present, amongst them being Sir Richard Quain, Sir William Broadbent, Sir William MacCormac, and many other well-known members of the medical profession. The toast of the evening was proposed by Lord Reay, and speeches were made by Sir Henry Thompson, Dr. Champneys, and others.

We have often spoken of the good work done by the Meath Home of Comfort for epileptic women and girls. We are glad that the movement is progressing in every way. The annual report (the first issued, we believe) is a most interesting and cheering record. Fifty-four inmates now reside in the home. Committee, medical superintendent—all devote their talents and energy to the task of making the lot of the patients better in every way. A governess has been added to the staff, and the industries of the home are carried on with interest and pride by the inmates Lady Meath has given another proof of her generous interest in the scheme by presenting the home with an additional wing, which will allow of the establishment of an isolation ward. The chapel, an earlier gift of the foundress, has been rendered of special interest by the confirmation of thirteen patients within its walls. Space does not allow us to dwell any longer on the many interesting points in the work and its progress, but we refer our readers to the admirably drawn up and interesting report of the home.

The Samaritan Fund of the Westminster Hospital is in urgent need of assistance, and an appeal is made to friends of the sick poor for help to carry on its beneficent work. By means of the Samaritan Fund upwards of 400 patients are sent every year to convalescent homes in order that they may regain their strength after severe operations or serious illness, and assistance in many other ways is given to those in need of it, the almoner being able to ascertain during his visitation of the sick the precise form of help which is best suited to each individual case. Unfortunately, the prevailing sickness, which will undoubtedly increase the calls upon the Samaritan Fund, will also tend to diminish its resources. One kind friend, who has annually given a reading by which upwards of £100 has been realised, is unable to render this help in consequence of illness involving a long absence abroad. The Rev. H. J. Fase, chaplain and almoner of the Samaritan Fund, or the secretary of the Hospital, will be very happy to supply further information or to receive any contributions which those who are interested in this most charitable work may be pleased to forward to either of them.

